# Rationale and design of a multicenter, randomized phase II trial of durvalumab with or without multitarget tyrosine kinase inhibitor as maintenance treatment in extensive‐stage small‐cell lung cancer patients (DURABLE study)

**DOI:** 10.1111/crj.13715

**Published:** 2023-11-10

**Authors:** Bo Zhang, Hua Zhong, Chunlei Shi, Zhiqiang Gao, Runbo Zhong, Aiqin Gu, Weimin Wang, Tianqing Chu, Liwen Xiong, Wei Zhang, Huimin Wang, Xueyan Zhang, Baohui Han

**Affiliations:** ^1^ Department of Respiratory and Critical Care Medicine Shanghai Chest Hospital, Shanghai Jiao Tong University School of Medicine Shanghai China

**Keywords:** anti‐angiogenesis, check‐point inhibitors, ES‐SCLC, maintenance treatment

## Abstract

**Introduction:**

Durvalumab is a check‐point inhibitor against programmed death ligand‐1 (PD‐L1), and anlotinib is a new orally administered multitarget tyrosine kinase inhibitor (TKI). Both agents have been approved in China. Preclinical and clinical trials have suggested that antiangiogenic therapy has the potential to alleviate immunosuppression and showed synergetic effect when combined with ICIs. However, it is unclear that whether this combination is effective when initiated as maintenance treatment in ES‐SCLC patients.

**Methods:**

This is a multicenter, randomized, phase II study. A total of 64 eligible patients who do not experience disease progression after four cycles platinum‐based chemotherapy combined with durvalumab will be randomized to durvalumab with anlotinib or durvalumab alone until disease progression, withdrawal of consent, or unacceptable toxicity. The primary endpoint is PFS (from randomization); secondary endpoint was OS and PFS (from diagnosis), objective response rate (ORR); disease control rate (DCR) and duration of response (DOR), safety and tolerability assessed by the National Cancer Institute Common Terminology Criteria for Adverse Events (CTCAE) version 5.0.

**Discussion:**

We conduct a phase II study to investigate the safety and efficacy of durvalumab combined with anlotinib as maintenance treatment in ES‐SCLC patients.

## 1 INTRODUCTION

Lung cancer has been the most common cancer in the world for several decades, accounting for 11.6% of the total new cases in 2018, and was also the leading cause of cancer‐related death (18.4% of the total cancer deaths).^1^ Small‐cell lung cancer (SCLC) represents approximately 13%–15% of all newly diagnosed lung cancers.^2^ SCLC is characterized by a highly aggressive disease with its rapid doubling time, high growth fraction and early metastasis.^2^ The prognosis of extensive‐stage small cell lung cancer (ES‐SCLC) is very poor. The median OS was 7 months, and only less than 7% of patients remain alive at 5 years after diagnosis.^3^

Four to six cycles of platinum‐based chemotherapy have been the standard care for patients with ES‐SCLC for the past 25 years.^4^, ^5^ Until the advent of check‐point inhibitors (ICIs), immunotherapy has been integrated into the clinical management of these patients. CASPIAN study suggested that the addition of durvalumab to first‐line chemotherapy resulted in significantly longer overall survival (OS) than chemotherapy alone.^6^ The updated results of this study sustained OS improvement versus platinum‐etoposide.^7^ In addition, IMpower‐133 study also demonstrates that chemotherapy combined with another programmed death ligand‐1 (PD‐L1) inhibitors‐atezolizumab, can confer survival benefit.^8^, ^9^

Anti‐angiogenesis agents are important treatment strategy in non‐small cell lung cancer when initiated as first‐line or maintenance treatment.^10^, ^11^ But series clinical trials have demonstrated that bevacizumab^12^, ^13^ and other anti‐angiogenesis treatment including rh‐endostatin,^14^ sunitinib^15^ and pazopanib^16^ failed to show survival benefit in SCLC patients. In addition, maintenance treatment with nivolumab plus ipilimumab did not prolong OS in these patients.^17^ Thus, consolidating maintenance therapy represents a largely unmet need.

Anlotinib (AL3818) is a new orally administered tyrosine kinase inhibitor that targets vascular endothelial growth factor receptor (VEGFR), fibroblast growth factor receptor (FGFR), platelet‐derived growth factor receptors (PDGFR) and c‐kit.^18^ In a randomized, phase II trial, anlotinib showed longer PFS (4.1 vs. 0.7 m hazard ratio [HR] 0.19, *p* < 0.0001) and OS (7.3 vs. 4.9 m; HR = 0.53, *p* = 0.0029) compared with placebo when administered as third‐ or further‐line treatment, thus has been approved as standard treatment in these patients.^19^

Both preclinical and clinical trials have suggested that antiangiogenic therapy has the potential to alleviate immunosuppression via inhibiting Treg differentiation and promoting the maturation of dendritic cells, thus showing synergetic effect.^20^, ^21^ Based on these results, we proposed our hypothesis that durvalumab combined with anlotinib may serve as a potential maintenance treatment in ES‐SCLC patients (Durvalumab Combined with Anlotinib as Maintenance Treatment In Extensive Stage Small Lung Cancer Patients (DURABLE)).

## 2 METHOD

### 2.1 Study design

**FIGURE 1:**
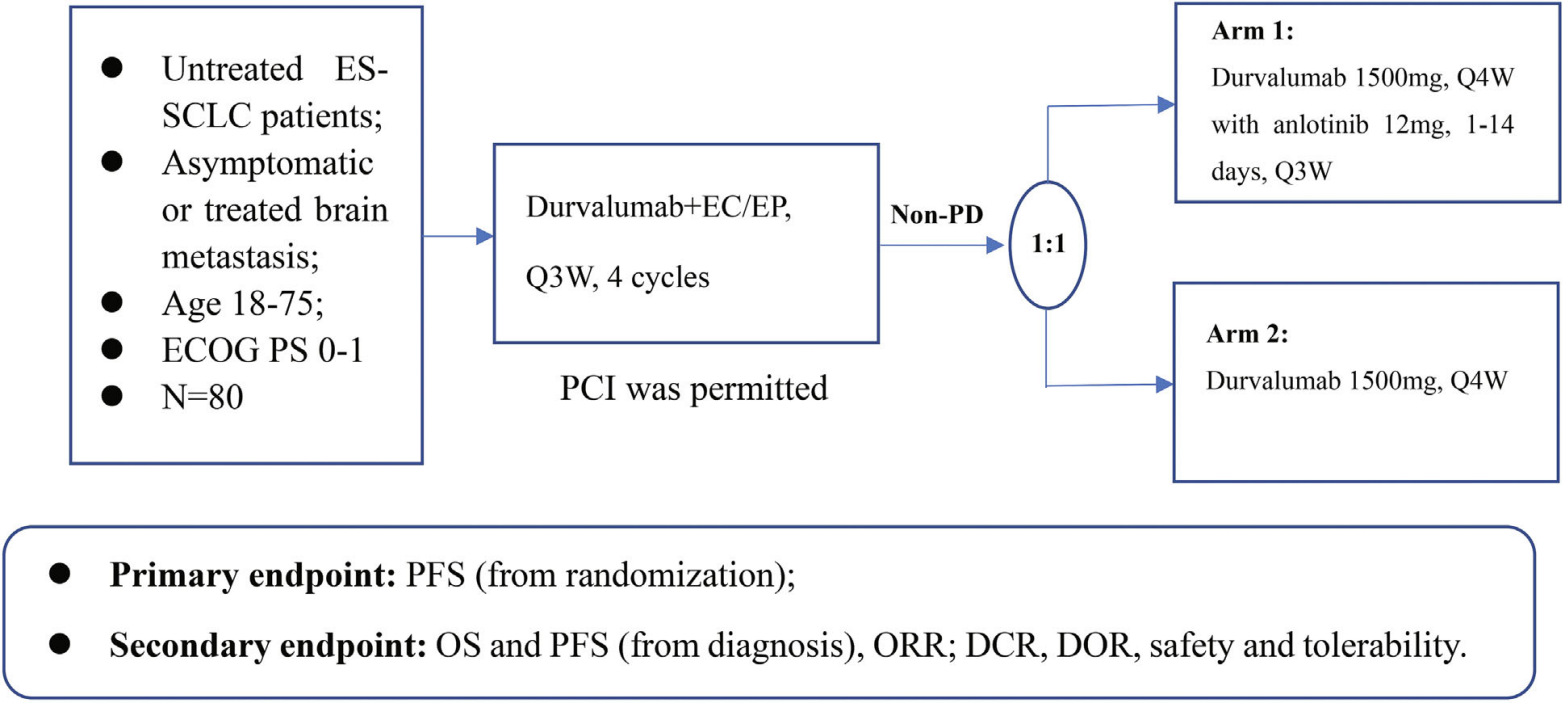
Study flow chart.

This is an open‐label, randomized, multicenter, phase II study. Eligible patients will be treated with durvalumab combined with up to four cycles of etoposide and platinum‐based chemotherapy (EP/EC). Patients who complete the four cycles of durvalumab plus EP/EC treatment and do not have progressive diseases (non‐PD patients) will be randomized in a 1:1 ratio to receive maintenance treatment durvalumab with anlotinib (Arm 1) or durvalumab alone (Arm 2) until confirmed PD, unacceptable toxicity or withdraw of written consent. Prophylactic cranial irradiation (PCI) is allowed at the investigators' discretion (Figure 1). Patients will attend a safety follow‐up visit 90 days after the last dose of durvalumab. The first eligible patient has been recruited at the end of September 2021. This study has been registered with clinicaltrial.gov, and the registration number is NCT04985851. The study was conducted in accordance with the Declaration of Helsinki. The study protocol and informed consent documents were approved by the ethical committees of Shanghai Chest Hospital (IS2150). Written informed consent is obtained from all participants.

### 2.2 Research point

The primary endpoint of the study was to assess the efficacy of durvalumab with anlotinib as maintenance therapy in terms of median progression‐free survival (PFS, from randomization); secondary endpoint was OS and PFS (from diagnosis), objective response rate (ORR); disease control rate (DCR) and duration of response (DOR), safety and tolerability assessed by the National Cancer Institute Common Terminology Criteria for Adverse Events (CTCAE) version 5.0. The exploratory endpoint of the study was to investigate potential biomarker based on peripheral blood and tumour tissue.

### 2.3 Inclusion criteria

Only patients who meet all of the following criteria will be enrolled in this study:

1. age ≥18 years and ≤75 years;
2. histologically or cytologically documented extensive disease (American Joint Committee on Cancer Stage [8th edition] IV SCLC [T any, N any, M1 a/b/c]), or T3–4 due to multiple lung nodules that are too extensive or have tumour/nodal volume that is too large to be encompassed in a tolerable radiation plan;
3. brain metastases must be asymptomatic or treated and stable. Patients with suspected brain metastases at screening should have a CT/MRI of the brain prior to study entry;
4. having at least one measurable lesion according to RECIST 1.1;
5. no prior systemic therapy for ES‐SCLC;
6. suitable to receive a platinum (cisplatin or carboplatin) based chemotherapy regimen as first‐line treatment for the ES‐SCLC;
7. ECOG PS score: 0 to 1;
8. life expectancy ≥12 weeks;
9. body weight >30 kg;
10. no prior exposure to immune‐mediated therapy including, but not limited to, other anti‐CTLA‐4, anti‐PD‐1, anti‐PD‐L1 and anti‐programmed cell death ligand 2 (anti‐PD‐L2) antibodies, excluding therapeutic anticancer vaccines;
11. no prior exposure to anti‐angiogenesis drugs;
12. adequate organ and marrow function as defined below:

- absolute neutrophil count ≥1.5 × 10^9^/L, platelet count ≥100 × 10^9^/L, haemoglobin ≥9.0 g/dL (no blood transfusion or no erythropoietin dependence within 7 days before enrollment);
- biochemical test results should meet the following criteria: serum bilirubin ≤1.5 times the upper limit of normal value (ULN); ALT and AST ≤2.5 *×* ULN; in case of liver metastases, ALT and AST ≤5 × ULN; creatinine clearance (CCr) >60 mL/min for patients on cisplatin and >45 mL/min for patients on carboplatin, as determined by Cockcroft‐Gault (using actual body weight).

13 international normalized ratio (INR) and prothrombin time (PT) ≤1.5 times ULN for patients not receiving therapeutic anticoagulation;
14 women of child‐bearing age should agree to take contraceptive measures (such as intrauterine devices, contraceptives or condoms) during the study and within 6 months after the study; non‐breast‐feeding patients whose serum or urinary pregnancy test should be negative; male patients should agree to take contraceptive measures during the study and within 6 months after the study; and
15 patients are voluntarily enrolled into the study, sign the informed consent form and have good compliance.

### 2.4 Exclusion criteria

Patients who meet any of the following criteria will be excluded:

1. patients who were diagnosed with mix SCLC or limited stage SCLC;
2. patients who have been treated with systemic therapy;
3. patients with inability to take oral medication, including but not limited to dysphagia, gastrointestinal resection, chronic diarrhoea and intestinal obstruction;
4. patients with any history of radiotherapy to the chest prior to systemic therapy or planned consolidation chest radiation therapy. Radiation therapy outside of the chest for palliative care (i.e., bone metastasis) is allowed but must be completed before the first dose of the study medication;
5. patients with major surgical procedure (as defined by the investigator) within 28 days prior to the first dose of study drugs;
6. patients with a history of allogeneic organ transplantation;
7. patients who have autoimmune disease, requiring systemic treatment (systemic steroids or immunosuppressive agents);
8. patients with active or prior documented autoimmune or inflammatory disorders, including inflammatory bowel disease, diverticulitis with the exception of diverticulosis, systemic lupus erythematosus, sarcoidosis syndrome or Wegener syndrome;
9. patients with active infection including tuberculosis, hepatitis B virus (known positive HBV surface antigen [HbsAg] result), hepatitis C virus (HCV) or human immunodeficiency virus (positive HIV 1/2 antibodies). Patients with a past or resolved HBV infection (defined as the presence of hepatitis B core antibody [anti‐HBc] and absence of HbsAg) are eligible. Patients positive for HCV antibody are eligible only if polymerase chain reaction is negative for HCV RNA;
10. patients with current or prior use of immunosuppressive medication within 14 days before the first dose of durvalumab. The following are exceptions to this criterion: intranasal, inhaled, topical steroids or local steroid injections (e.g., intra articular injection); systemic corticosteroids at physiologic doses not to exceed 10 mg/day of prednisone or its equivalent. Steroids as premedication for hypersensitivity reactions (e.g., CT scan premedication);
11. patients with a history of active primary immunodeficiency;
12. patients who are known to have symptomatic brain metastases or carcinomatous meningitis, or brain or leptomeningeal disease diagnosed by CT or MRI at the time of screening;
13. patients with receipt of live, attenuated vaccine within 30 days prior to the first dose of study drugs;
14. patients with metastatic disease that involves major airways or blood vessels, or centrally located mediastinal tumour masses (<30 mm from the carina) of large volume;
15. patients with evidence of bleeding diathesis or significant coagulopathy (in absence of therapeutic anticoagulation);
16. patients who do not have completely controlled eye inflammation or eye infection, or any condition that may lead to the above‐mentioned ocular diseases;
17. patients with any severe and/or unable to control diseases, including:

- patients with interstitial lung disease;
- patients with blood pressure unable to be controlled ideally (systolic pressure >140 mmHg, diastolic pressure >90 mmHg);
- patients with Grade 1 or higher myocardial ischemia, myocardial infarction or malignant arrhythmias (including QTc ≥450 ms [male], QTc ≥470 ms [female] and patients with Grade 1 or higher congestive heart failure; NYHA classification);
- patients with cirrhosis, decompensated liver disease or active hepatitis;
- patients with poorly controlled diabetes (fasting blood glucose >10 mmol/L);
- patients with serious chronic gastrointestinal conditions associated with diarrhoea;
- patients with urine protein ≥++, and 24‐h urinary protein excretion >1.0 g confirmed;
- patients with psychiatric illness/social situations that would limit compliance with study requirement, substantially increase risk of incurring AEs or compromise the ability of the patient to give written informed consent;

18 patients with uncontrolled hypercalcemia (>1.5 mmol/L calcium ion or calcium >12 mg/dL or corrected serum calcium >ULN), or symptomatic hypercalcemia requiring continued diphosphate therapy;
19 patients with unhealed wounds or fractures;
20 patients with arterial or venous thromboembolic events occurred within 6 months, such as cerebrovascular accident (including transient ischemic attack), deep vein thrombosis and pulmonary embolism;
21 patients who are known to have severe allergies (≥Grade 3) to active ingredients and any excipients of anlotinib or durvalumab;
22 patients who have other malignant tumours (except radical cervical carcinoma in situ, non‐melanoma skin cancer, etc.) at the same time;
23 patients who are the subjects or their sexual partners cannot or refuse to take effective contraceptive measures during the clinical trial;
24 patients who are pregnant or breast‐feeding women; and
25 patients in other situations who are evaluated by the investigator to be ineligible to be enrolled.

### 2.5 Randomization, treatment, dose and assessment

#### 2.5.1 Randomization

Non‐PD patients who completed four cycles of durvalumab with chemotherapy will be randomized into maintenance phase. Once the patient is confirmed to be eligible to enter maintenance phase, the investigator or suitably trained delegate will obtain a unique randomization number via the interactive voice response system or interactive web response system (IVRS/IWRS). The system will randomize the eligible patient to one of the two treatment arms.

#### 2.5.2 Treatment, dose and assessment

Durvalumab 1500 mg will be administered for all patients concurrently with chemotherapy every 3 weeks, starting on Week 0 for four cycles. Non‐PD patients who completed the four cycles of the combination treatment will be randomized 1:1 into Arm 1 or Arm 2. Eligible patients should begin continuous treatment in 3 days after randomization. In Arm 1, durvalumab 1500 mg monotherapy will be continued every 4 weeks. Anlotinib will be orally administered 12 mg once a day, 14 days‐on and 7 days‐off. Anlotinib is available at three dose levels, 12, 10 and 8 mg. Patients in Arm 2 will be treated with durvalumab monotherapy. Dose reduction may take place whenever toxicity is not controlled with optimal supportive care during the study. If a subject subsequently tolerates treatment well at that level in the judgement of the investigator, the dose may be increased to the next dose level. If a subject cannot tolerate treatment after dose reduction to 8 mg, treatment will be discontinued. Efficacy assessment will be performed every 6–8 weeks. After discontinuation of study treatment, patients will continue to be followed for survival via phone calls or email every 3 months to study end. The survival status (including cause of death) and the date of death or last follow‐up date will be collected.

### 2.6 Rationale for setting the number of enrolled participants

The study will randomize approximately 64 eligible patients 1:1 to durvalumab with anlotinib and durvalumab monotherapy group. The primary analysis of PFS (from randomization) will occur when approximately 50 progression events have been observed in the 64 randomized patients. If the true PFS HR for the comparison of durvalumab plus anlotinib versus durvalumab is 0.6 (5 vs. 3 m), 50 progression events will provide 70% power to demonstrate a statistically significant difference in PFS at a 20% two‐sided significance level. And assuming about 20% patients will experience disease progression in the first four cycles treatment (from CASPIAN) and 5% patients will lose of follow‐up, the overall sample size is 80.

### 2.7 Population to be analysed

Efficacy will be analysed based on the intention‐to‐treat (ITT) population and per‐protocol set (PPS). Safety will be analysed based on safety analysis set (SAS).

ITT: all randomly assigned patients.
PPS: all the participants in the ITT except patients with violation of inclusion/exclusion criteria or violation for prohibited concomitant drugs/therapies.
SAS: patients who received at least one dose of study drugs.

### 2.8 Statistical methods

Baseline characteristics, incidence and severity of adverse events will be summarized. The number and percent of subjects achieving objective responses (CR or PR) will be summarized along with corresponding two‐sided 95% CI using binomial distribution. Median PFS and OS will be calculated based on the Kaplan–Meier method and compared by log‐rank test. Cox proportional hazard regression model will be used to identify independent prognostic factors.

## 3 DISCUSSION

The advent and administration of ICIs have revolutionized the first‐line treatment in ES‐SCLC patients.^6^, ^8^ Despite the ORR is encouraging, but the survival benefit is not sustained. Thus, efficient treatment of these patients still represents a formidable challenge.

Anlotinib monotherapy has been approved as third‐ or further‐line treatment in ES‐SCLC patients.^19^ The synergetic effect of antiangiogenic agent and ICIs support the combination strategy as maintenance treatment. To the best of our knowledge, this is the first study to investigate the safety and efficacy of durvalumab with anlotinib as maintenance treatment in these patients.^20^, ^21^ The first patient has been enrolled at the end of September 2021 and is expected to take 36 months.

## 4 CONCLUSION

The results of the DURABLE study will provide new data about safety and efficacy about multitarget tyrosine kinase inhibitor as maintenance treatment in ES‐SCLC patients.

## AUTHOR CONTRIBUTIONS

All authors had full access to the protocol of the study and take responsibility for the integrity of the protocol. *Conceptualization*: Baohui Han, Hua Zhong, Bo Zhang and Chunlei Shi. *Investigation*: Bo Zhang, Hua Zhong, Chunlei Shi, Zhiqiang Gao, Runbo Zhong, Aiqin Gu, Weimin Wang, Tianqing Chu, Liwen Xiong, Wei Zhang, Huimin Wang, Xueyan Zhang and Baohui Han. *Methodology*: Baohui Han, Hua Zhong and Bo Zhang. *Supervision*: Hua Zhong and Baohui Han. *Writing—original draft preparation*: Bo Zhang, Hua Zhong, Chunlei Shi, Zhiqiang Gao, Runbo Zhong, Aiqin Gu and Weimin Wang. W*riting—review and editing*: Tianqing Chu, Liwen Xiong, Wei Zhang, Huimin Wang, Xueyan Zhang and Baohui Han.

## CONFLICT OF INTEREST STATEMENT

None.

## ETHICS STATEMENT

The study will be conducted in accordance with the Declaration of Helsinki. The study protocol and informed consent documents were approved by the ethical committees of Shanghai Chest Hospital (IS2150). Written informed consent is obtained from all participants.

## Data Availability

Data sharing not applicable to this article as no datasets were generated or analysed during the current study.
